# Selective activation of the hypothalamic orexinergic but not melanin-concentrating hormone neurons following pilocarpine-induced seizures in rats

**DOI:** 10.3389/fnins.2022.1056706

**Published:** 2022-12-01

**Authors:** Zhenquan He, Xiao Wang, Kang Ma, Leyi Zheng, Yan Zhang, Chunhong Liu, Tao Sun, Peng Wang, Weifang Rong, Jianguo Niu

**Affiliations:** ^1^Key Laboratory of Craniocerebral Diseases of Ningxia Hui Autonomous Region, Ningxia Medical University, Yinchuan, China; ^2^Department of Anatomy, School of Basic Medical Sciences, Ningxia Medical University, Yinchuan, China; ^3^Department of Paediatrics, Yinchuan Maternal and Child Health Care Hospital, Yinchuan, China; ^4^Department of Neurology, General Hospital of Ningxia Medical University, Yinchuan, China; ^5^Department of Anatomy and Physiology, Shanghai Jiao Tong University School of Medicine, Shanghai, China

**Keywords:** epilepsy, orexin neurons, sleep-wake cycle, hypothalamus, amygdala

## Abstract

**Introduction:**

Sleep disorders are common comorbidities in patients with temporal lobe epilepsy (TLE), but the underlying mechanisms remain poorly understood. Since the lateral hypothalamic (LH) and the perifornical orexinergic (ORX) and melanin-concentrating hormone (MCH) neurons are known to play opposing roles in the regulation of sleep and arousal, dysregulation of ORX and MCH neurons might contribute to the disturbance of sleep-wakefulness following epileptic seizures.

**Methods:**

To test this hypothesis, rats were treated with lithium chloride and pilocarpine to induce status epilepticus (SE). Electroencephalogram (EEG) and electromyograph (EMG) were recorded for analysis of sleep-wake states before and 24 h after SE. Double-labeling immunohistochemistry of c-Fos and ORX or MCH was performed on brain sections from the epileptic and control rats. In addition, anterograde and retrograde tracers in combination with c-Fos immunohistochemistry were used to analyze the possible activation of the amygdala to ORX neural pathways following seizures.

**Results:**

It was found that epileptic rats displayed prolonged wake phase and decreased non-rapid eye movement (NREM) and rapid eye movement (REM) phase compared to the control rats. Prominent neuronal activation was observed in the amygdala and the hypothalamus following seizures. Interestingly, in the LH and the perifornical nucleus, ORX but not MCH neurons were significantly activated (c-Fos^+^). Neural tracing showed that seizure-activated (c-Fos^+^) ORX neurons were closely contacted by axon terminals originating from neurons in the medial amygdala.

**Discussion:**

These findings suggest that the spread of epileptic activity from amygdala to the hypothalamus causes selective activation of the wake-promoting ORX neurons but not sleep-promoting MCH neurons, which might contribute to the disturbance of sleep-wakefulness in TLE.

## Introduction

Epilepsy is one of the most common neurological disorders, which affects millions of people globally. Sleep disorders, such as altered sleep architecture, daytime sleepiness, and sleep-disordered breathing, are common comorbidities in patients with epilepsy (Wang et al., 2018; Sudbrack-Oliveira et al., 2019). Indeed, sleep and epilepsy are interrelated bidirectionally, in that not only epileptic seizures disrupt sleep architecture, but also disordered sleep can lead to increased seizures and may exacerbate the progression of neuronal damage (Im et al., 2016; Bazil, 2017; Peter-Derex et al., 2020). A better understanding of the neurological basis underlying the bidirectional interaction between epilepsy and sleep is important for effective management of patients with epilepsy.

Lesions in the temporal lobe, such as those in the hippocampus, cortex, or amygdala, are the most common causes of epileptic seizures (McDonald and Mott, 2017; Tilelli et al., 2021; Feinstein et al., 2022). Temporal lobe epilepsy (TLE) often causes disruptions in sleep. For example, Bazil et al. (2000) studied a cohort of 34 patients and found that temporal lobe complex partial seizures decrease rapid eye movement (REM) sleep, which may be partly responsible for the prolonged impairment of functioning that some patients report following seizures. Yildiz et al. (2015) reported that TLE patients have higher risk of obstructive sleep apnea (OSA) than patients with extratemporal lobe epilepsy. The specific neuronal pathways responsible for the disordered sleep in TLE are yet to be identified. We suspect aberrant activation of the neural pathways from amygdala to hypothalamus might be involved. Amygdala damage is often observed in TLE patients and epileptic animal models, including the pilocarpine hydrochloride-induced epileptic rat (Scholl et al., 2013; Matovu and Cavalheiro, 2022). The amygdala project extensively and may transmit seizure activity to many other brain regions, including the hypothalamus, which plays key roles in the switch between sleep and wakefulness (Weera et al., 2021; Kaplan et al., 2022).

Two distinct populations of peptidergic neurons, the orexin (ORX) and melanin-concentrating hormone (MCH) neurons in lateral hypothalamic (LH) and the perifornical nucleus are known to exert opposing influences on sleep and arousal (Konadhode et al., 2015; Hung et al., 2020). ORX and MCH neurons are intermingled in LH and the perifornical region and they share similar target areas in the brain (Concetti and Burdakov, 2021). ORX neurons are wake-active and their activation promotes transition from sleep to wakefulness (De Luca et al., 2022). In contrast, MCH neurons are sleep-active and their activation initiates and maintains sleep (Monti et al., 2013). ORX level increases in epileptic patients and animal models and intracranial administration of ORX increases seizure activity (Kortunay et al., 2012; Goudarzi et al., 2015; Riahi et al., 2015; Arslan et al., 2022), whilst administration of MCH inhibits seizures in epileptic animal models (Parks et al., 2010; Clynen et al., 2014). Previous reports suggested that ORX and MCH neurons might receive direct inputs from the amygdala (Niu et al., 2012; González et al., 2016). These data have led us to hypothesize that the amygdala may transmit epileptic activity to LH and perifornical region, resulting in dysregulation of ORX and MCH neurons and consequent sleep disruption in TLE. To test the hypothesis, c-Fos (hereafter referred to as Fos) and ORX or MCH immunofluorescent staining was conducted on the rat brain following pilocarpine hydrochloride-induced seizures. In addition, neural tracing combined with Fos staining was carried out in order to understand the connection between amygdala and the ORX neurons.

## Materials and methods

### Animals

Adult male Sprague–Dawley (SD) rats weighing between 250 and 300 g were used in this study. All rats were singly housed in an air-conditioned room (22 ± 1°C) with a 12/12 h light/dark cycle (lights on at 8:00 and lights off at 20:00). Animals were allowed access to food and water *ad libitum*. The study has been carried out in accordance with the governmental regulations on the use of experimental animals with prior approval by the Ethics Committee of Ningxia Medial University (Document #IACUC-NYLAC-2020-030).

### General protocol

*Experiment 1* to analyze sleep-wakefulness following pilocarpine-induced seizures: rats (*n* = 6) were first implanted with electroencephalogram (EEG) and electromyograph (EMG) electrodes and were allowed to recover in their home-cages for 1 week. Then rats were transferred to the recording chamber and were allowed to habituate to the chamber environment for 2 days followed by EEG and EMG recording for 24 h. The rats then received successive injections of lithium chloride and pilocarpine to induce seizures. Twenty-four hours after termination of seizure, EEG and EMG were again recorded for 24 h. EEG and EMG signals were used to analyze the sleep-wakefulness states before and after pilocarpine-induced seizures.

*Experiment 2* to analyze neuronal activation in amygdala and the hypothalamus: rats (*n* = 6) received successive injections of lithium chloride and pilocarpine to induce seizures. Control rats (*n* = 6) were injected with equal volumes of saline. One hour after the first seizure occurred, the animal was sacrificed and the brain collected for analysis of neuronal activation in amygdala and the hypothalamus through immunostaining for Fos, ORX, and MCH.

*Experiment 3* to analyze the connection between amygdala and ORX neurons: A total of 26 rats were used in the experiment to investigate the connection between medial amygdala and ORX neurons. Twenty rats were first injected with the retrograde tracer cholera toxin B subunit (CTb) in LH (*n* = 10) or the perifornical nucleus (*n* = 10). One week later, half of the rats received successive injections of lithium chloride and pilocarpine to induce seizures and were euthanized 1 h after the first seizure. The other half without induction of seizure were also euthanized to serve as control. The brains were harvested for Fos and CTb immunohistochemistry. Another six rats were injected with a mixture of CTb and biotinylated dextran amine (BDA, for anterograde tracing) in the medical amygdala. They received successive injections of lithium chloride and pilocarpine to induce seizures and were euthanized 1 h after the first seizure. The brains were harvested for immunofluorescent staining of CTb/BDA, ORX, and Fos to investigate the possible reciprocal connections between medial amygdala and ORX neurons.

### Induction of seizures

Epileptic seizures were induced in the rat as previously described (Smolensky et al., 2019; Zuo et al., 2020), with slight modifications. Briefly, rats were first administered 130 mg/kg lithium chloride intraperitoneally (i.p.). A total of 18–20 h later, seizure was induced by i.p. injection of 30 mg/kg pilocarpine. Thirty minutes prior to pilocarpine administration, 2 mg/kg scopolamine (i.p.) was applied to each rat to reduce the peripheral effects of pilocarpine. Seizure activity was scored by Racine’s scale (Racine, 1972). Animals that developed class 3 (forelimb clonus), class 4 (rearing), and class 5 (rearing and falling) seizure activity within 60 min, were considered to be in status epilepticus (SE). SE was terminated by diazepam (10 mg/kg i.p.) 30 min after the first seizure activity occurred.

### Implantation of electroencephalogram and electromyograph electrodes

Rats were anesthetized with i.p. injection of ketamine (100 mg/kg, Phoenix, USA) and xylazine (10 mg/kg, Sigma) and placed onto a stereotaxic frame. Two pairs of EEG screw electrodes were implanted into the skull (two in the frontal bones at AP = 1 mm and ML = ±3.0 mm and two in the parietal bones at AP = −4.0 mm, and ML =±3.0 mm on each side). Two flexible EMG wire electrodes (A-M systems, USA) were inserted into bilateral trapezoid muscle in the neck. The free ends of the EEG/EMG electrodes leads were inserted into a head socket and then fixed on the skull with dental cement. Upon completion of the procedure, rats were allowed to recover on a warm blanket until awakened from anesthesia and were then placed back to their home cages.

### Electroencephalogram/electromyograph recording and analysis of sleep-wake states

In *Experiment 1*, 24 h EEG and EMG signals were collected before and 24 h after termination of pilocarpine-induced seizures. EEG and EMG signals were amplified (A-M systems, USA) and filtered (EEG band pass filtered at 0.75–20 Hz and EMG low pass filtered at 20 Hz). The signal was then digitized at a sampling rate of 256 Hz and recorded with time-lock video on a PC using SleepSign 3.0 software (Kissei Comtec, Japan). Wake-sleep states were manually scored in 10 s epochs based on the digitized EEG/EMG of each rat. Wakefulness was identified by the presence of desynchronized-EEG and high-EMG activity. Non-rapid eye movement (NREM) sleep was identified by the presence of high-amplitude, slow-wave EEG and low-EMG activity relative to that of waking. Rapid-eye-movement (REM) sleep was identified by the presence of regular theta activity on EEG, coupled with low-EMG activity relative to that of NREM sleep.

### Microinjection of neural tracers

For microinjection of neural tracers (*Experiment 3*), rats were anesthetized with i.p. injection of ketamine (100 mg/kg, Phoenix, USA) and xylazine (10 mg/kg, Sigma) and then mounted onto a stereotaxic frame with the skull plane in the horizontal position. The skull was shaved and a longitudinal cut was made on the skin to expose the cranium. The bregma was used as the reference point for placement of the Hamilton micro-syringe. A 1–2 mm hole was drilled on the cranial bone and the micro-syringe filled with neural tracers was advanced to LH (3.0 mm caudal, 1.8 mm lateral, and 8.7 mm ventral to the bregma), the perifornical nucleus (2.4 mm caudal, 1.2 mm lateral, and 8.6 mm ventral to the bregma) or the medial amygdala (2.2 mm caudal, 3.6 mm lateral, and 9.4 mm ventral to the bregma). Twenty rats (10 control and 10 later treated with pilocarpine) were injected with 50 nl of 0.2% CTb (LIST, USA) in the LH or the perifornical nucleus for retrograde labeling of amygdala neurons projecting to LH or perifornical nucleus. Another six rats (later treated with pilocarpine) were injected with 100 nl of a mixture of CTB (0.2%) and BDA (10%, Thermo Fisher Scientific, USA) in the medial amygdala to label the possible reciprocal connections between amygdala and ORX neurons.

### Brain tissue preparation and immunohistochemistry/immunofluorescence

Rats were deeply anesthetized and perfused transcardially with 500 ml of saline, followed by 500 ml of 4% Paraformaldehyde in 0.1 M PBS. The brains were removed and post-fixed in paraformaldehyde (4% in PBS) at room temperature for 4–6 h, and then immersed in cold sucrose solution (30% in PBS) until the brain sank to the bottom. Subsequently, the brains were cut into 40 μm serial sections using a cryostat. The sections were collected in parallel into six groups and stored in preservation solution. Sections were then processed for immunohistochemistry or immunofluorescence as needed.

In *Experiment 2*, neuronal activation following pilocarpine-induced seizures was studied by immunohistochemical staining of Fos. Brain sections containing the hypothalamus and the amygdala were incubated with mouse anti-Fos (1:400, Santa Cruz Biotechnology, USA) at 4°C overnight. After washing three times with PBS, sections were incubated with biotinylated donkey anti-mouse IgG (1:200, Jackson ImmunoResearch, USA) for 3 h at room temperature. After washing three times with PBS, sections were incubated with the avidin-biotin-peroxidase complex (ABC, 1:200, Vector, USA) for 1 h. Finally, sections were incubated with diaminobenzidine (DAB, 1:20, Zhongshanjinqiao, Beijing, China) containing 0.06% ammonium nickel sulfate and 5% cobaltous chloride for 10 min at room temperature. The sections were mounted on slides and observed under a microscope for inspection of Fos-immunoreactivity (black).

Double immunofluorescent staining for Fos and ORX or MCH was conducted to study the possible activation of hypothalamic ORX and MCH neurons following pilocarpine-induced seizures. Sections containing hypothalamus were blocked within 1% BSA (Sigma) in PBS containing 0.2% triton X-100 for 1 h, and then incubated with mouse anti-Fos (1:400) and rabbit anti-ORX/MCH (1:800, Phoenix Pharmaceuticals Inc., USA) at 4°C overnight. Sections were washed three times with PBS and incubated within CyTM5-conjugated donkey anti-mouse IgG and CyTM3-conjugated donkey anti-rabbit IgG (both 1:200, Jackson ImmunoResearch, USA) at room temperature for 3 h. After washing three times with PBS, the sections were mounted on slides and observed under fluorescence microscope.

In *Experiment 3*, double immunohistochemical staining for Fos and the retrograde tracer CTb (pre-injected to LH or perifornical nucleus) were conducted to study the activation of amygdala neurons with projections to LH or perifornical nucleus following pilocarpine-induced seizures. After Fos was immunostained following the steps described earlier, the sections were incubated with rabbit anti-CTb (1: 500, List Biological Labs, USA) at 4°C overnight. After three washes with PBS, the sections were incubated with biotinylated donkey anti-rabbit IgG (1: 200, Jackson ImmunoResearch, USA) at room temperature for 3 h. After another three washes with PBS, sections were incubated with ABC (1: 200, Vector, USA) for 1 h. Finally, the sections were incubated with DAB (1: 20, Zhongshanjinqiao, Beijing, China) at room temperature for 10 min. The sections were observed under the microscope for Fos (black) and CTb (brown) immunoreactivity in the amygdala.

Triple immunofluorescent staining for Fos, ORX and CTb or BDA (pre-injected to the medial amygdala) was conducted to study the projections between amygdala and seizure-activated ORX neurons. Brain sections were incubated with mouse anti-Fos (1:400), rabbit anti-ORX (1:800) and goat anti-CTb (1:500, List Biological Labs, USA), or only mouse anti-Fos and rabbit anti-ORX (to detect BDA) at 4°C overnight. Sections were washed three times with PBS and then incubated within CyTM5-conjugated donkey anti-mouse IgG (1:200, Jackson ImmunoResearch, USA, to detect Fos), CyTM3-conjugated donkey anti-rabbit IgG (1:200, Jackson ImmunoResearch, USA, to detect ORX), and Alexa fluor 488-conjugated donkey anti-goat IgG (1:200, Jackson ImmunoResearch, to detect CTb) or CyTM2-conjugated Streptavidin (1:200, Jackson ImmunoResearch, USA, to detect BDA) at room temperature for 3 h. After three washes with PBS, sections were mounted on slides and observed under the microscope (Leica, DM6B, Germany).

### Data quantification and statistical analysis

In Experiment 1, rats that failed to complete all the tests due to death or electrode shedding were not processed for Fos immunohistochemistry. In Experiments 2 and 3, rats with inaccurate injection sites for CTB and/or BDA were also excluded from quantitative analysis. The number of animals included in each analysis is specified in figure legends. For quantification of immunoreactive neurons, images were optimized in brightness and contrast using Adobe Photoshop and the number of Fos^+^ cells or CTb^+^ cells in regions of interest were counted manually using the Photoshop’s counting tool and their distribution was mapped using Adobe Illustrator 2020. Three representative sections of each rat were counted. Statistical analyses were performed using GraphPad Prism 9.0. Numerical data are reported as the mean ± SEM. Unpaired or paired Student’s *t*-test was used to compare between two groups. To compare multiple groups, two-way ANOVA was used. A *P*-value of less than 0.05 was considered as indicating statistically significant differences.

## Results

### Sleep disturbance occurred in rats following pilocarpine-induced seizures

**FIGURE 1:**
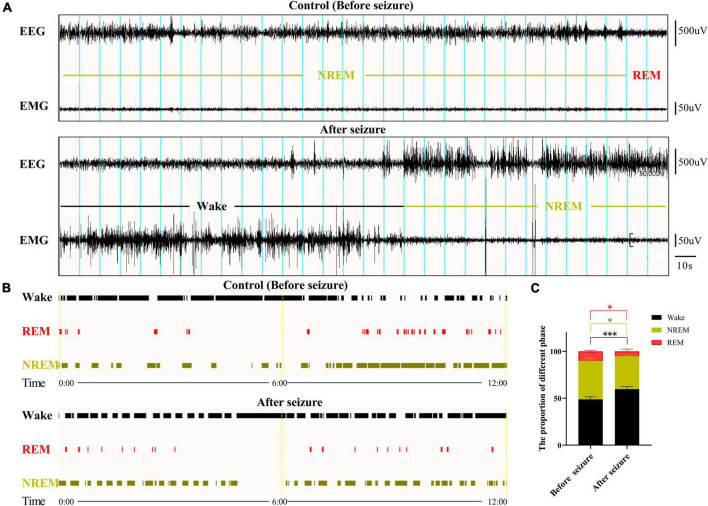
Sleep-wake cycle was altered following pilocarpine-induced seizures. **(A)** Sleep-wake cycle was analyzed based on 24 h EEG and EMG signals collected before and after pilocarpine-induced status epilepticus (SE). Shown here are two 5 min EEG and EMG signals at 10 a.m. from a rat before and after SE, respectively. Blue lines indicate 10 s epochs. The sleep-wake state for each 10 s epoch was designated as in **(B)**. **(C)** Bar graph showing that rats displayed increased wake state and decreased REM and NREM state 1 day after SE. *P < 0.05, ^***^P < 0.001, *n* = 3, two-way ANOVA.

Sleep-wakefulness analysis was performed based on the 24 h EEG and EMG signals collected before and after seizure. Wakefulness was characterized by the presence of low amplitude, desynchronized EEG (theta and alpha waves) coupled with high EMG activity. NREM sleep was identified by the presence of high-amplitude delta waves on EEG, coupled with low EMG activity relative to that of wakefulness. REM sleep was identified by the presence of regular theta waves on EEG, coupled with low EMG activity relative to that of NREM sleep. Inspections of the EEG and EMG data showed generally increased amplitude of EEG and EMG activity (Figure 1A). The sleep-wakefulness analysis revealed a significant increase in wakefulness (control vs. seizure group: 48.81 ± 2.7 vs. 59.64 ± 2.71%, *P* = 0.0009, *n* = 3 rats in each group), a significant decrease in NREM sleep (control vs. seizure group: 40.89 ± 2.72 vs. 35.29 ± 2.24%, *P* = 0.0252, *n* = 3 rats in each group), and a significant decrease in REM sleep (10.29 ± 0.98 to 5.08 ± 2.41%, *P* = 0.0345, *n* = 3 rats in each group) 24 h after induction of seizures (Figures 1B,C).

### Prominent neuronal activation in hypothalamus and amygdala following seizures

**FIGURE 2:**
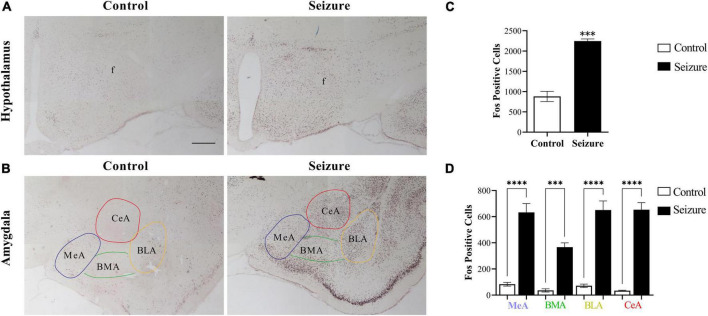
Pilocarpine-induced status epilepticus led to neuronal activation in hypothalamus and amygdala. **(A)** Representative images of Fos immunohistochemical staining in the hypothalamus of a control and an epileptic rat; **(B)** representative images of Fos staining in the amygdala of a control and an epileptic rat. Bar: 500 μm in **(A)**, which is also applicable in **(B)**. **(C,D)** Bar graphs show the number of Fos^+^ cells in hypothalamus and amygdala in control (non-epileptic) and epileptic rats. ^***^P < 0.001, ^****^P < 0.0001, Student’s *t*-test **(C)** or two-way ANOVA **(D)**, *n* = 3 rats for each group. BLA, basolateral amygdaloid nucleus, anterior part; BMA, basomedial amygdaloid nucleus, anterior part; CeA, central amygdaloid nucleus, anterior part; f, fornix; MeA, medial amygdaloid nucleus, anterior part.

To test the hypothesis that amygdala may transmit epileptic activity to LH and perifornical nucleus, we first examined neuronal activation in the amygdala and hypothalamus following seizures through Fos immunohistochemistry. In the hypothalamus, a small number of Fos^+^ cells were observed in control rats, and the number of Fos^+^ cells was dramatically increased in seizure rats (Figure 2A). Cell counting in two representative coronal sections from each rat indicated that the number of Fos^+^ cells (2,245 ± 53.54) in seizures rats (*n* = 3) was significantly higher than in control rats (880.7 ± 125.8, *n* = 3) (Figure 2C).

Similarly, Fos^+^ cells were significantly increased in the amygdala in seizure rats (4,250.0 ± 1.45, *n* = 3) than in control rats (556.7 ± 8.82, *n* = 3) (Figure 2B). We then counted the Fos^+^ cells in four amygdaloid subnuclei, the anterior part of basolateral amygdaloid nucleus (BLA), medial amygdaloid nucleus (MeA), central amygdaloid nucleus (CeA), and anterior part of basomedial amygdaloid nucleus (BMA) separately. The results indicated significantly more neuronal activation in these subnuclei in seizure rats (BLA: 650.7 ± 69.87; MeA: 633.0 ± 67.57; CeA: 653.7 ± 53.70; BMA: 367.0 ± 32.70, *n* = 3) compared with the control rats (BLA: 71.33 ± 12.99; MeA: 83.33 ± 14.44; CeA: 34.33 ± 3.18; BMA: 36.0 ± 12.01, *n* = 3) (Figures 2B,D).

### Orexin neurons but not melanin-concentrating hormone neurons were activated in lateral hypothalamic and perifornical nucleus of seizure rats

**FIGURE 3:**
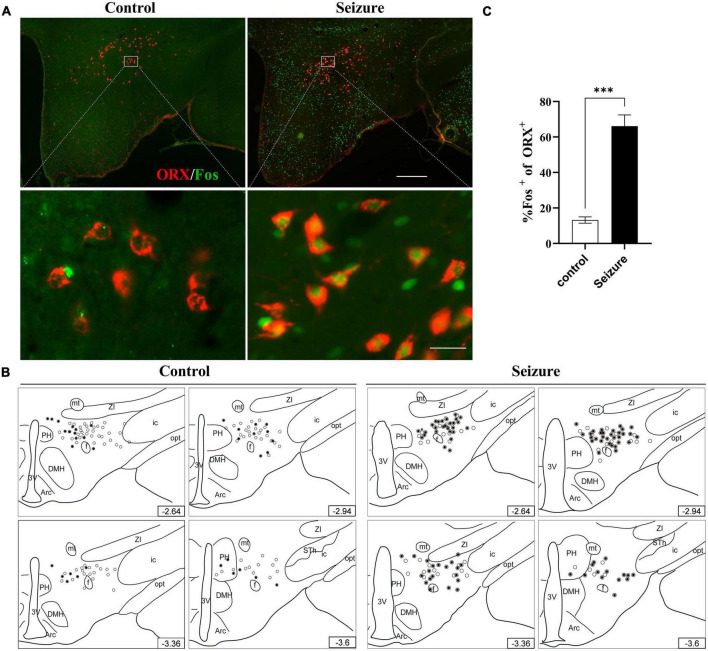
Hypothalamic ORX neurons were activated in seizure rats. **(A)** Representative images of double immunofluorescent staining for ORX (red) and Fos (green) in the hypothalamus of a control and a seizure rat; bar: 500 μm in the upper panel and 30 μm in the lower panel. **(B)** The distribution of Fos^+^ (filled circle) or Fos^–^ ORX neurons (clear circle) in 4 rostral-caudal sections of hypothalamus of control and seizure rats, with each clear circle representing 3 Fos^–^ ORX neurons and each filled circle representing 3 Fos^+^ ORX neurons. The number on the lower right of each section indicates the distance from the bregma; **(C)** bar graph showing the proportion of ORX neurons that were activated (Fos^+^). ^***^P < 0.001, *n* = 4 rats for each group, Student’s *t*-test. 3V, third ventricle; Arc, arcuate hypothalamic nucleus; DMH, dorsomedial hypothalamic nucleus; f, fornix; ic, internal capsule; mt, mammillothalamic tract; opt, optic tract; PH, posterior hypothalamic nucleus; STh, subthalamic nucleus; ZI, zona incerta.

**FIGURE 4:**
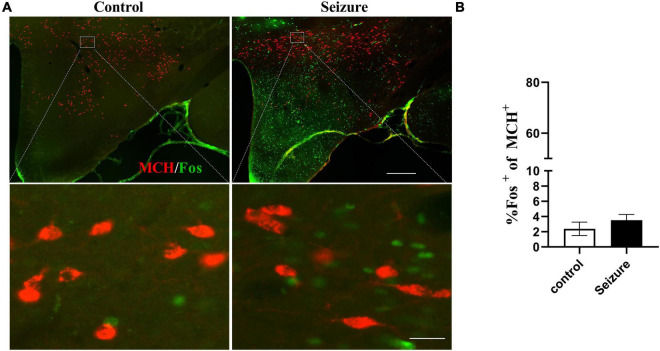
Pilocarpine-induced seizures did not cause significant activation of MCH neurons. **(A)** Representative images of MCH (red) and Fos (green) immunofluorescent staining in the hypothalamus of control or seizure rats; bar: 500 μm in the upper panel and 30 μm in the lower panel. **(B)** Bar graph showing the proportion of MCH neurons that were Fos^+^. *n* = 4 rats for each group, Student’s *t*-test.

We then conducted double immunofluorescent staining to investigate the possible activation of ORX and MCH neurons in LH and the perifornical nucleus following seizures. As exemplified in Figure 3A, only a minority of ORX^+^ neurons in LH and perifornical nucleus were Fos^+^ in control rats, whereas a large proportion of ORX^+^ neurons were Fos^+^ in seizure rats. The distribution of ORX and Fos immunofluorescence in four rostro-caudal sections of the hypothalamus was illustrated by line drawings (Figure 3B). Counting of double-stained cells indicated prominent activation of ORX neurons (66.12 ± 6.31%) in seizure rats (*n* = 4) than in control rats (13.18 ± 1.77%, *n* = 4) (Figure 3C). In contrast, double immunofluorescent staining for MCH and Fos indicated that Fos expression was rare in MCH^+^ neurons in control rats and the proportion of Fos^+^ MCH neurons were not significantly different in seizure rats compared with the control rats (3.51 ± 0.75 vs. 2.39 ± 0.88%, *P* = 0.369, *n* = 4 each) (Figures 4A,B). Therefore, seizures seemed to cause selective activation of wake-promoting ORX neurons but not sleep-promoting MCH neurons, which was in line with our hypothesis that dysregulation of ORX and MCH neurons might underlie sleep disturbance following seizures.

### Amygdala neurons might transmit epileptic activity to orexin neurons in lateral hypothalamic and perifornical nucleus

**FIGURE 5:**
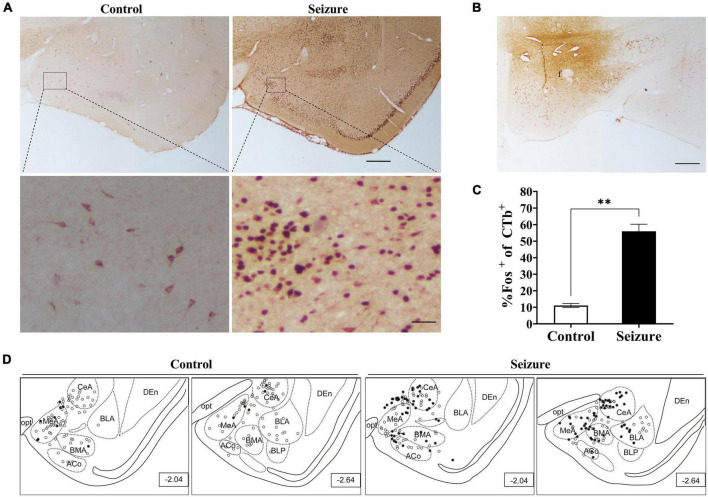
Pilocarpine-induced statis epilepticus (SE) caused significant activation of amygdaloid neurons projecting to the perifornical region of the hypothalamus. **(A)** Representative images of Fos (dark purple to black) and CTb (brown) immunostaining in the amygdala in a control and a pilocarpine-treated (seizure) rat pre-injected with CTb in the perifornical region **(B)**. Bar: 500 μm in upper panels of **(A)** and in **(B)**, and 60 μm in lower panels of **(A)**. **(C)** Bar graph showing the proportions of CTb-labeled amygdala neurons that were activated (Fos^+^) in control and seizure rats. ^**^P < 0.01, *n* = 2 rats for each group, Student’s *t*-test. **(D)** The distribution of Fos^+^ or Fos^–^ CTb-labeled neurons in 4 rostral-caudal sections of the amygdala in control and seizure rats, with each clear circle representing 1 Fos^–^ CTb-labeled neuron and each filled circle representing 1 Fos^+^ CTb-labeled neuron. The numbers on the right lower corner of each sections indicate the distance from the bregma. ACo, anterior cortical amygdaloid nucleus; BLA, basolateral amygdaloid nucleus; BLP, basolateral amygdaloid nucleus, posterior part; BMA, basomedial amygdaloid nucleus, anterior part,; CeA, central amygdaloid nucleus; DEn, dorsal endopiriform nucleus; f, fornix; MeA, medial amygdaloid nucleus, anterior part; opt, optic tract.

**FIGURE 6:**
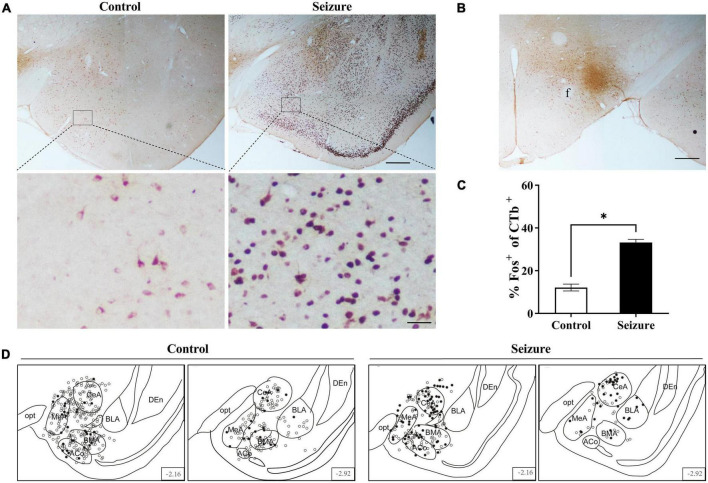
Pilocarpine-induced statis epilepticus (SE) caused significant activation of amygdaloid neurons projecting to the lateral hypothalamus (LH). **(A)** Representative images of Fos (dark purple to black) and CTb (brown) immunostaining in the amygdala in a control and a pilocarpine-treated (seizure) rat pre-injected with CTb in LH as shown in **(B)**. Bar: 500 μm in upper panels of **(A)** and in **(B)**, and 60 μm in lower panels of **(A)**. **(C)** Bar graph showing the proportions of CTb-labeled amygdala neurons that were activated (Fos^+^) in control and seizure rats. *P < 0.05, *n* = 2 rats for each group, Student’s *t*-test. **(D)** The distribution of Fos^+^ or Fos^–^ CTb-labeled neurons in 4 rostral-caudal sections of the amygdala in control and seizure rats, with each clear circle representing 1 Fos^–^ CTb-labeled neuron and each filled circle representing 1 Fos^+^ CTb-labeled neuron. The numbers on the right lower corner of each section indicate the distance from the bregma. ACo, anterior cortical amygdaloid nucleus; BLA, basolateral amygdaloid nucleus; BMA, basomedial amygdaloid nucleus, anterior part; CeA, central amygdaloid nucleus; DEn, dorsal endopiriform nucleus; f, fornix; MeA, medial amygdaloid nucleus, anterior part; opt, optic tract.

We injected the retrograde neuronal tracer CTb to the perifornical nucleus or LH to examine the possibility that the amygdala might transmit epileptic activity to ORX neurons. Figure 5 shows examples of CTb and Fos staining in the amygdala in a control and a pilocarpine-induced seizure rat injected with CTb in the perifornical region (Figure 5B). CTb-labeled neurons could be detected in the amygdala ipsilateral to the injection site (Figure 5A). There was no significant difference in the number of CTb-labeled amygdala neurons between control rats (113.5 ± 19.25/section, *n* = 2 rats) and seizure rats (118.3 ± 2/section, *n* = rats). However, significantly higher proportion of Fos^+^ neurons were co-labeled with CTb in seizure rats (55.98 ± 4.25%, *n* = 2) than in control rats (11.12 ± 1.25%, *n* = 2) (Figure 5C). Additionally, it was noticed that CTb and Fos co-labeled neurons were concentrated in medial subnuclei (e.g., BMA, MeA, and CeA) in seizure rats (Figure 5D). Similarly, in rats injected with CTb in LH (Figure 6B), CTb-labeled neurons were detected in the ipsilateral amygdala (Figure 6A) and a higher proportion of Fos^+^ neurons were co-labeled with CTb in the medial amygdala in seizure rats (33.21 ± 1.49%, *n* = 2) than in control rats (12.12 ± 1.61%, *n* = 2 rats) (Figures 6C,D). These results suggested that neurons in the medial amygdala which project to LH/perifornical nucleus were activated following seizures.

**FIGURE 7:**
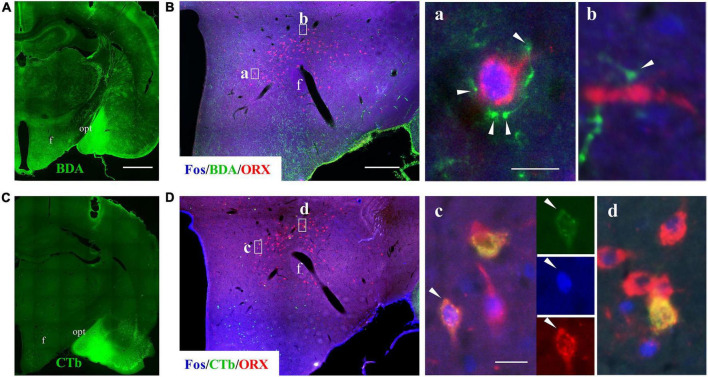
Pilocarpine-induced SE caused activation of reciprocal connections between medial amygdala and ORX neurons. Panels **(A,C)** show staining for BDA and CTb (which was co-injected into medial amygdala) in two serial coronal sections from a seizure rat. **(B)** A representative image of triple immunofluorescent staining for Fos (blue), BDA (green), and ORX (red) in the hypothalamus of a rat subjected to pilocarpine-induced SE, with squared areas (a,b) enlarged in the right panels. Note that BDA-labeled fibers and terminal boutons encircle a Fos^+^/ORX^+^ soma (a) or in close contact with ORX^+^ axon (b). **(D)** A representative image of triple immunostaining for Fos (blue), CTb (green), and ORX (red) in the hypothalamus of the same rat, with squared areas enlarged in the right panels. Note that some seizure-activated (Fos^+^) ORX neurons were labeled with CTb. Bar: 1,000 μm in **(A,C)**, 400 μm in **(B,D)**, and 20 μm in a–d. f, fornix; opt, optic tract.

We then sought for evidence that neurons in the medial amygdala project directly to seizure-activated ORX neurons. To this end, BDA (anterograde tracer) and CTb (retrograde tracer) were co-injected into the medial amygdala in rats later treated with lithium chloride/pilocarpine (Figures 7A,C) and the hypothalamus were immunostained for Fos, ORX and BDA or CTb. It was found that seizure-activated (Fos^+^) ORX neurons were closely contacted by BDA-labeled fibers or terminal boutons (Figure 7B). Interestingly, we noticed that many seizure-activated (Fos^+^) ORX neurons were also labeled by the retrograde tracer CTb (Figure 7D). These results suggested that reciprocal monosynaptic projections exist between neurons in medial amygdala and ORX neurons in LH and perifornical nucleus and activation of these reciprocal neuronal pathways might contribute to sleep disturbance in epileptic seizures.

## Discussion

Patients with epilepsy often endure severe sleep disruption and sleep disorders may exacerbate epilepsy, but the neurobiological basis for the bidirectional interaction between sleep and epilepsy is still not very clear (Wang et al., 2018; Gibbon et al., 2019). ORX neurons in the LH and perifornical region are well-known to promote arousal and wakefulness, whilst MCH neurons in the same regions promote the transition to sleep (Ono and Yamanaka, 2017). The current study has provided evidence that epileptic seizures may cause selective activation of ORX neurons but not MCH neurons. Our data also revealed reciprocal connections between medial amygdala and ORX neurons, suggesting that activation of the reciprocal neural pathways might underlie the sleep disruption following epileptic seizures.

The pilocarpine model is the most widely used rodent model of TLE (Curia et al., 2008; Lévesque et al., 2021). Sleep fragmentation was previously reported in rats with pilocarpine-induced chronic epilepsy (single i.p. dose of 360 mg/kg pilocarpine; Matos et al., 2010). In the present study, rats were treated with 130 mg/kg lithium chloride (i.p.) and 30 mg/kg pilocarpine (i.p.) to induce SE, which was terminated by diazepam 30 min after the first seizure activity occurred. EEG and EMG analysis showed that 24 h after termination of pilocarpine-induced SE, rats exhibited prolonged wakefulness and decreased REM and NREM sleep. The results corroborate with the findings of Matos et al. (2010) and confirm 30 min of pilocarpine-induced SE may cause disruptions in sleep-wake cycle.

We conducted Fos staining to label neurons activated by seizures. Not surprisingly, we found widespread neuronal activation in amygdala and the hypothalamus following pilocarpine-induced SE. The hypothalamus is known to play critical roles in regulation of sleep-wakefulness. Specifically, two groups of peptidergic neurons in the lateral hypothalamus and perifornical region, i.e., the ORX and MCH neurons, are known to play opposing roles in regulation of wakefulness and sleep (Hassani et al., 2009; Jones, 2020). ORX neurons are wake-active and administration of ORX led to increased arousal state and decreased sleep state in rats (Cun et al., 2014; Mavanji et al., 2015). Moreover, loss of ORX neurons or mutations of ORX or ORX receptor genes are associated with narcolepsy in humans, mice and dogs (Tonokura et al., 2007; Nepovimova et al., 2019) and there has been evidence suggesting that decreased ORX neurotransmission might be associated with sleep problems in the elderly (Hunt et al., 2015). In contrast to the wake-promoting role of ORX neurons, MCH neurons are sleep-active and intracranial administration of MCH increases REM and NREM sleep, whilst administration of MCH receptor antagonist causes disruption in REM and NREM sleep and increases in arousal (Ahnaou et al., 2008; Tsunematsu et al., 2014; Naganuma et al., 2018; Sharma et al., 2018). In the current study, it was found that ORX but not MCH neurons were preferentially activated (Fos^+^) following 30 min of pilocarpine-induced SE. The results were in line with our hypothesis that dysregulation of ORX and MCH neurons probably underlies the disrupted sleep-wake cycle following seizures.

The amygdala plays an indispensable role in epilepsy (Tilelli et al., 2021). The amygdala has extensive local and distant projections, hence may transmit epileptic activity to its inner nuclei and other brain regions, including the hypothalamus (Weera et al., 2021). Direct projections from amygdala to the lateral hypothalamus and ORX neurons have been demonstrated previously (Niu et al., 2012; González et al., 2016; Reppucci and Petrovich, 2016), but it is unknown whether these projections are activated in epileptic seizures. In the current study, CTB-labeled Fos^+^ neurons were detected in the ipsilateral amygdala, particularly in the medial subnuclei (CeA, MeA, and BMA), in epileptic rats injected with the retrograde tracer CTb in perifornical region or LH. In epileptic rats co-injected with BDA and CTb in the medial amygdala, seizure-activated (Fos^+^) ORX neurons were found to be closely contacted by BDA-labeled fibers or terminal boutons, suggesting that the medial amygdala might transmit epileptic activity to ORX neurons through the monosynaptic connections, leading to activation of ORX neurons following seizures. MCH neurons were not activated in the epileptic rats. Whether this was because the monosynaptic inputs from medial amygdala preferentially target ORX neurons but not MCH neurons is unknown.

An interesting finding from this set of experiments was that seizure-activated (Fos^+^) ORX neurons were also retrogradely labeled with CTb which was co-injected with BDA in the medial amygdala. The hypothalamic ORX neurons are known to project to many brain regions, including the amygdala (Peyron et al., 1998; Bisetti et al., 2006). Our data tend to suggest that there exist reciprocal, probably monosynaptic connections between medial amygdala and hypothalamic ORX neurons. Two subtypes of ORX receptors, OX1R and OX2R, have been identified and the amygdaloid complex is enriched with the excitatory OX1R but not OX2R (Lu et al., 2000; Grafe and Bhatnagar, 2018). These data provide clues for the bidirectional interaction between TLE and sleep, in that transmission of epileptic activity from medial amygdala to hypothalamus may cause activation of wake-promoting ORX neurons, which may in turn cause recurrent activation of the medial amygdala. Further studies using electrophysiological and opto-/chemogenetic approaches are required to test this hypothesis.

## Conclusion

In summary, the present study has demonstrated selective activation of the wake-promoting ORX neurons but not the sleep-promoting MCH neurons in the lithium/pilocarpine-induced epileptic rats. Additionally, the study has provided preliminary evidence that activation of reciprocal connections between the medial amygdala and ORX neurons might underlie the disrupted sleep-wake cycle seen in this rat model of TLE.

## Data availability statement

The raw data supporting the conclusions of this article will be made available by the authors, without undue reservation.

## Ethics statement

The animal study was reviewed and approved by the Ethics Committee of Ningxia Medial University.

## Author contributions

JN and WR conceived and designed the experiments, interpreted the data, and critically revised the manuscript. ZH, XW, KM, LZ, and YZ performed the experiments and acquired the data. CL, TS, and PW analyzed the data. ZH drafted the manuscript. All authors read and approved the final version of the manuscript.
